# The Hospital

**Published:** 1917-10-06

**Authors:** 


THE
### INSTITUTIONAL WORKER
Being a Special Supplement to "The Hospital"

### OUR BUREAU OF INFORMATION.

### Rules for Correspondents.

1. Every letter must be accompanied by the coupon to be cut from the back cover (inside page) of THE HOSPITAL, current issue, and must contain the name and address of the correspondent with pseudonym for publication if desired. Replies by post cannot be given save under exceptional circumstances at the Editor's discretion.

2. Letters from Approved Homes in reply to special needs published in the Bureau should state terms and full particulars, and be sent prepaid under cover to the Editor of the Bureau with name written across coupon for identification.

3. Proprietors of Homes which have not yet been entered on the List of Approved Homes, but have spare accommodation likely to suit special needs, are invited to write for an application form for registration The fee for registration, which includes two announcements of the Home in the Bureau and other privileges, is 10s.

4. All communications to be addressed to the Editor of THE HOSPITAL, 28 Southampton Street, Strand, London, W.C. 2, and marked "Bureau of Information."

### INSTITUTION FACTS AND FIGURES.

### Starting a Venereal Block.

#### Answer to September Question.

By M. K. S.

THE question was :

**It is proposed to start a venereal block, containing two wards of twelve beds each (male and female), a clinic, and an out-patients' department. (a) What nursing staff will be required, and how should it be distributed? (b) What equipment will be necessary for each department?**

Just at the present time the question of starting a new department for venereal cases is one of great interest, as most of the general hospitals have been invited and have responded to the appeal to start a clinic and equip beds.

The clinic would be, of course, run through the out-patient department, the only special staff being the medical man in charge, who would be assisted by the existing nursing staff of the out-patient department. There would be special days for male and female cases, an advertisement to this effect being inserted in the local paper which reaches all districts from which the hospital receives patients. Whenever possible, the sister or staff nurse would be in attendance for the female patients, a male attendant being present for male patients.

THE CLINIC EQUIPMENT.

At least three rooms fitted with sinks, hot and cold water, sterilisers, cupboards for lotions and specimens, lotion-bottles, a table for examination purposes, electric hand-lights, and special stands for the administering of 606 and saline would be necessary. Also at least six couches with pillows and rugs on which the patients after injection could remain until fit to go home. Several screens would also be required, and specially marked dust-bins into which all soiled linen could be placed for disinfection before being removed to the laundry.

The medical and nursing staff would require gowns distinctively marked, and all linen used for this department should have a distinguishing and clearly read mark, so that it would not in any way be mixed with the linen of other departments.

Special instruments, douche-cans, rubber gloves, bowls, etc., should also be of a distinctive colour and kept in their own department.

THE NURSING STAFF.

In the early stages of a provincial clinic two fully-trained nurses and one male assistant ought to be sufficient. The clinics would probably be held three or four times in the week, and should be so arranged that they should in no way interfere with the routine work of the out-patient department.

As the patients suffer mainly from faintness after the actual operation of injection, it is a case of resting on couches under the supervision of the sister that is required.

For a block of twenty-four beds, which is evidently meant to cover a very large area or a thickly-populated town, one fully-trained, experienced sister would be required, assisted by a staff nurse on night duty and two staff nurses on day duty; it is better to have only fully trained nurses for this work, at least in the early days of the experiment. When possible, one male nurse from the men's wards should be employed. The women's ward would necessarily include cots for "large babies," and probably those cases whose diseases are the result of venereal trouble would also be treated there instead of in the general wards of the hospital as heretofore.

Patients of this type would be unlike the ordinary hospital cases, and the utmost tact would be required in their treatment and management.

THE WARD EQUIPMENT.

The equipment, which would include all ward furniture, bedding, and utensils with the exception of instruments, which would be under the province of the medical officer, should for a ward of twenty-four beds include twenty-four bedsteads of the usual hospital pattern, easy to move and clean; twenty-four regulation ward lockers, which act as a bed-table for meals, dressings, etc.; twenty-four chairs for use of the visitors; one stool for the purpose of stripping the bed when bed-making is in progress. At least four comfortable chairs and four couches, a ward-table, clocks, footstools, bed-boards; the usual dressing-wagon with lotion-bottles, etc., a test-table with fixtures, an oxygen-stand, hot-water tins, hand-lamps, and a well-stocked ward library which contains plenty of up-to-date, interesting literature.

Bedding required for a ward of this size would be 100 sheets, 100 draw-sheets, 24 bed mackintoshes (two sizes), 30 quilts, 30 bed-jackets, 12 special shirts, 40 bath-towels, 80 blankets, 30 mattress covers, 6 tablecloths, 30 locker-cloths, 24 rubbers of various kinds; Dressing-gowns and getting-up blankets, 40 pillows and some cushions with washing covers will also be needed, and for wards of this kind 6 screens with washing covers apiece would be required. The surgeons and nurses would also require gowns and rubber gloves. All these articles should be made of special material or clearly marked, so that they would run no risk of being mixed with the hospital linen.

Lavatory and kitchen utensils should be plentiful and of material that can be easily cleansed.

GENERAL OBSERVATIONS.

It is difficult in these early days to lay down hard-and-fast rules about the management of a venereal block, especially as they are in embryo as yet, and the time is not ripe to explain special methods. It seems very necessary to interest the patients, when possible, in light ward tasks, and much will depend on the atmosphere of the ward, which largely lies in the hands of the Sister who controls it. A middle-aged dependable woman should preferably attend to the domestic duties. Fortunately, most hospitals have such labour already to hand, and those hospital workers who are interested in this new venture will look forward with great interest to its evolution.

### QUESTION for OCTOBER.

### AN ISOLATION HOSPITAL DIFFICULTY.

In an isolation hospital wards are often closed for months at a time. In spite of good ventilation and dry weather, the enamel of the bedsteads peels off, and the stoves get rusty very quickly. Explain fully what can be done to prevent this.

### RULES.

**The following rules must be observed:—**

1. Contributions must be written on one side of the paper. Brevity and terseness are desirable features. The MSS. must bear the name and address of the sender and be accompanied by coupon to be cut from the back cover (inside page) of the current issue of The Hospital. A pseudonym must be chosen if the name is not to be published.

2. Contributions must be addressed to the Editor of The Hospital, Institutional Worker Supplement, 28 & 29 Southampton Street, Strand, London, W.C. 2, should reach him before the end of the current month, and be marked in the left-hand corner "Facts and Figures."

**A minimum payment of Half-a-Guinea will be made for each published answer.**

### ENQUIRIES AND ANSWERS.

### *EMPLOYMENT AND TRAINING.*

**Massage and Electrical Treatment.**

You cannot do better than take out a course of instruction at the National Hospital and University College Hospital School of Massage and Electrical Treatment, 29 and 30 Queen Square, W.C. 1. In addition to class teaching, students receive instruction in the out-patient and special departments of the National Hospital and the University College Hospital. The fee for a twenty-four weeks' course in massage is £15 15s., and for a twelve weeks' course in medical electricity £5 5s. There is a hostel in connection with the school available for women students. The course commences this month. Apply to the Secretary.—Thorough.

**Midwifery Training.**

You do not say where you wish to train; there are many recognised midwifery training-schools throughout the country. The Central Midwives Board holds examinations six times a year in London and three times a year in the provinces. The provincial centre nearest your home would probably be Bristol. Lists of training-schools and approved teachers can be obtained upon application to the Secretary, Central Midwives Board, Caxton House, Westminster, S.W.—Nurse F. B.

### *MISCELLANEOUS.*

**Old X-Ray Plates.**

No hospital should permit the destruction of waste material without first of all considering the question of its possible use in other directions. Large quantities of glass are used in the *x*-ray department of hospitals every day, and many of the plates taken are discarded. These should be stored carefully and disposed of periodically. Their sale will produce a small return to the hospital, but more important still is the fact that their salvage will economise the labour expended in the manufacture of glass. Mr. Richard Shaw, of Royal Cottage, Royal Road, Ramsgate, purchases plates of all sizes from hospitals, and guarantees the destruction of the films. —L. S.

**Device for Moving Patients.**

A clever and simple device for lifting helpless patients from their beds is described by Dr. Collingwood Fenwick in the *Lancet*. It takes the form of a narrow canvas sheet, fitted with four handles on each side, which, having been passed under the patient, is lifted by two nurses or orderlies on each side. The patient, if being removed to the theatre, is then placed on a wheeled stretcher, to be lifted in the same way on to the operating table. These sheets are in use at the Military Hospital, Stepping Hill, Hazel Grove, near Stockport, and they were made by the village sadler at a cost of 6s. 6d. each. The method is obviously an improvement on the old plan of lifting by means of a sheet, is safer for the patient, and less tiring to the nurses or orderlies.

